# Application of array comparative genomic hybridization (array- CGH) for detection of chromosomal imbalances in children with developmental delay/congenital malformations in Saudi Arabia

**DOI:** 10.1186/1471-2164-15-S2-P52

**Published:** 2014-04-02

**Authors:** Ibtessam Ramzi Hussein, Adeel G Chaudhary, Randa Bassiouni, Maha AlQuaiti, Samira Sogaty, Mohammed Al-Qahtani

**Affiliations:** 1 Centre of Excellence in Genomic Medicine Research, King Abdulaziz University, Jeddah, KSA; 2 Faculty of Medical Sciences, King Abdulaziz University, Jeddah, KSA; 3 Pediatrics Dept., Ministry of Health, Al-Taif, Jeddah, KSA; 4 Maternity & Child Hospital, Jeddah, Jeddah, KSA

## Background

Micro**a**rray–based **C**omparative **G**enomic **H**ybridization (a-CGH) has enabled wide investigation of the genome at high resolution and has been implemented in different centers as a clinical diagnostic tool. Chromosomal imbalances are implicated in the etiology of Developmental Delay (DD)/Intellectual Disability (ID)/Congenital Malformations. However, most of these cases may not be diagnosed by conventional cytogenetic techniques. We aimed to establish a-CGH technique and assess its potential as a diagnostic tool for chromosomal imbalances and to detect known and novel chromosomal aberrations in patients with DD/ congenital malformations.

## Materials and methods

A total of 75 patients presented with DD/ congenital malformations with or without ID were referred to the CEGMR for cytogenetic analyses. We used both conventional cytogenetic G-banding and Fluorescent *in-situ* hybridization techniques (FISH), besides we applied (array-CGH) high resolution Agilent scanner with 1X244 K array format in 25 samples and 2X400 K format in 50 samples.

## Results

Chromosomal aberrations were detected in 10/72 (13.8%) patients by G-banding technique and 4/50 (8%) by FISH technique, however, 17/72 (23.6%) were diagnosed by a-CGH technique. All micro-deletion syndromes and partial duplications were detected by the chromosomal microarray technique: (Del 15 (q11.2); Del 15 (q13-14); Del 22 (q11.2); Del 7 (q11.23); Del 18 (q21 q23); Del 1 (p36); del 21q21-q23, del 13q21-q31.3, del 11q24.2-q25, and duplications in: dup 18p (p11.21); dup 15 (q11 q23); dup 18 (q23). However, one patient with unbalanced translocation could not be detected by this technique. The increase in the CNVs number detected by a-CGH needs further investigation for contribution to phenotypes.

## Conclusions

Our results indicate the strength of high resolution genomic arrays in diagnosing cases of unknown etiology and in detection of contiguous genomic alterations in the wide spectrum of cases with DD/ID/congenital malformations.
